# Supplementary material for: Using insects to detect, monitor and predict the distribution of Xylella fastidiosa: a case study in Corsica
Source: Sci Rep. 2018 Oct 23;8:15628. doi: 10.1038/s41598-018-33957-z (PMC6199265; doi:10.1038/s41598-018-33957-z)
Supplement: Supplementary file 1 — Supplementary data [file 41598_2018_33957_MOESM1_ESM.docx]

**Supplementary data to**

**Using insects to detect, monitor and predict the distribution of Xylella fastidiosa: a case study in Corsica**

Astrid Cruaud, Anne-Alicia Gonzalez, Martin Godefroid, Sabine Nidelet, Jean-Claude Streito, Jean-Marc Thuillier, Jean-Pierre Rossi, Sylvain Santoni and Jean-Yves Rasplus.

**Appendix 1. DNA extraction protocol optimised for the study.**

**Appendix 2. Precautions to limit the risk of contaminations for the nested PCR approach.**

**Appendix 3. References from which occurrences of *Philaenus spumarius* were compiled.**

**Figure S1. Life cycle of *Philaenus spumarius* in Corsica as presently understood.**

The life cycle of *P. spumarius* in Corsica needs to be more precisely investigated especially in a context of global changes. It is noteworthy that it may vary from year to year depending on weather conditions.

**Figure S2. Phylogenetic trees obtained from the concatenation of a) the loci classically included in the MLST of *Xf* and b) their reduced sequences obtained with the nested PCR approach.**

All genomes available in Genbank (last access October 19th 2017) were included in the analysis. Bootstrap supports are reported at nodes (RAxML, 1000 Bootstrap replicates, 1 partition per locus, GTR + Γ with 4 categories)

**Figure S3. Presence / absence maps of *Philaenus spumarius*** derived from the conversion of habitat suitability using (a) the lowest presence threshold or (b) the threshold value maximizing the sensitivity and the specificity. Areas where *P. spumarius* is considered to be present are depicted in red.

**Table S1. Sampling sites.**

Note: the 32 specimens tested for the presence of *Xf* in each sites are named as follows: codevial_0101 to codevial_0132 (e.g. specimens collected in site G are named JSTR03697_0101 to JSTR03697_0132)

**Table S2. Primers used for the nested PCR approach**

**Table S3. Nested PCR mastermix used for a single locus**

**Table S4. Nested PCR mastermix used to target multiple loci**

**Table S5. PCR conditions for PCR1, PCR1 Triplex and PCR2.**

**Appendix 1.**

The method for isolating DNA from insect to detect the presence of *Xf* was based on the protocol of Brady et al. 2011, with some modifications detailed below.

**Preparation of specimens and tissue disruption**

Specimens were getting out from ethanol with forceps, which were flame sterilized before use and between each use. Specimens were then dried on a clean paper towel.

A single insect was placed in a 2 mL grinding tube (MP Biomedicals, Lysing Matrix A, cat N° 116910050) and 135 µL of Lysis Buffer was added.

By series of 48, tubes were placed in a Mixer Mill MM 301 (Retsch GmbH, GER) tissue homogenizer, and samples were disrupted at room temperature for 2 minutes at 20 oscillations per second.

**Cell lysis**

In each tube, 10 µl of Ready Lyse Lysosyme and 10 µL of RNAse solution were added. Samples were homogenized by vortexing, and then incubated for 30 min at 37°C.

135 µL of 2X Extraction Buffer and 10 µL of Proteinase K solution were added. Samples were homogenized by vortexing, and then incubated 60 min at 56°C.

**DNA purification**

125 µL of cold Potassium acetate were added. Samples were homogenized by vortexing, incubated 10 min and lysates were cleared with centrifugation at 12000 rpm for 10 min at 4°C.

Supernatants were transferred into a KingFisher deep-well 96 plate (ThermoFisher Scientific, cat No 95040450) and 270 µL of Guanidium hydrochloride, 375 µL of Isopropanol and 15 µl of suspension of magnetic beads Chemagic (Perkin Elmer Chemagen, cat No CMG-252-A) were added.

The rest of the DNA purification was automated on the KingFisher Flex (ThermoFisher Scientific, cat No KingFisher™ Flex) instrument.

Four Deep-well 96 plates (ThermoFisher Scientific, cat No 95040450) that must be used with the KingFisher were prepared and placed in the following position.

- Position 1: 1 DeepWell plate 96 with 600 µL of Washing Solution 1
- Position 2: 1 DeepWell plate 96 with 600 µL of Washing Solution 1
- Position 3: 1 DeepWell plate 96 with 600 µL of 75 % ethanol
- Position 4:1 DeepWell plate 96 with 600 µL of 75 % ethanol

A 96 wells microplate (ThermoFisher Scientific, cat No 97002540) with 50 µL of TE Low 1X per well for the elution step of the purified DNA was placed last.

The purification was done by using the Software Protocol MagMAX DNA tissue cells of the KingFisher Flex Instrument.

**Composition of buffers**

Lysis Buffer: 10 mM Tris pH 8.0, 1 mM EDTA, 100 mM NaCl, 1% PVP 40000, 1% sodium bisulfite

Ready Lyse Lysosyme: 50 mM Tris pH 8.0, 0.1 mM EDTA, 100 mM NaCl, 20mM CaCl_2_, 0.1% TritonX100, 20 mg/mL lysozyme (Roche Diagnostics, cat n° 10 837 059 001, Lysozyme, dry powder, 23 000 U/mg), 50% glycerol.

RNase solution:  RNase A 0.1 mg/mL in ultrapure water (Roche Diagnostics, cat n°10109169001,RNase A, dry powder, 50 U/mg)

2X Extraction Buffer: 60 mM Tris pH 8.0, 60 mM EDTA, 10% Tween 20, 1% Triton X100, 1.6M Guanidium hydrochloride, 200mM NaCl, 6mM CaCl_2_, 1% PVP 40000, 1% sodium bisulfite.

Proteinase K solution: Qiagen, cat No 19133, Proteinase K > 600 mAU/ml.

Potassium acetate 3M/5M: for 100 mL mix 60 mL of 5 M potassium acetate, 11.5 mL of glacial acetic acid (37%), and 28.5 mL ultrapure water. The resulting solution is 3 M with respect to potassium and 5 M with respect to acetate.

Guanidium hydrochloride: 7.8 M in ultrapure water

Washing solution 1: 10 mM Tris pH 8.0, 0.1 mM EDTA, 60 mM potassium acetate, 65% ethanol.

TE Low 1X: 10 mM Tris pH 8.0, 0.1 mM EDTA

**Cited reference**

Brady, J.A., Faske, J.B., Castañeda-Gill, J.M., King, J.L., Mitchell, F.L., 2011. High-throughput DNA isolation method for detection of *Xylella fastidiosa* in plant and insect samples. J. Microbiol. Methods 86, 310-312.

**Appendix 2.**

The high sensitivity of nested PCRs makes the method prone to contamination, which can lead to inaccurate results. Carry-over contaminants from previous PCRs are the major sources of false positive results. The contaminants may be carried over from previous amplification reactions due to aerosols, contaminated pipettes, surfaces, gloves or reagents.

To prevent carry-over contaminants when amplifying DNA, though this offers no absolute guarantee we:

- used a separate lab for set-up and amplification,
- worked as cleanly as possible in a clean environment,
- minimized the number of pipetting steps,
- paid close attention when opening tubes or plates after amplification.

The different stages of the nested PCR protocol were carried out in different and dedicated rooms or spaces, with dedicated equipment, respecting the principles of unidirectional flow.

All pipetting was done using filter tips.

The most critical point to avoid carry-over contamination is the transfer of samples between PCR1 and PCR2. This is why we implemented the following strict procedure:

- The PCR1 and PCR2 amplifications were carried out in 96-well plates with only 24 samples, harmoniously distributed so that each full well was surrounded by empty wells and 2 full wells were never located side by side.

- The PCR plates were closed with a pierceable aluminum sealing film (Corning Inc, USA, Axygen Platemax, cat No: PCR-AS-200).

- At the end of PCR1, 5μL / 50μL of PCR product were transferred to a new plate for PCR2. The transfer was carried out with a Beckman FXp (Beckman Coulter Life Sciences, In, USA) pipetting robot by piercing the aluminum film. The speed of movement of the pipetting modules, according to the 3 axes, were set to the lowest possible speed.

- PCR1 products were not purified before PCR2 to reduce handling.

The following controls were used for PCR1 and PCR2:

Negative controls:

- 2 μg of phage Lambda DNA (ThermoFisher Scientific, cat No: SD0011)

- sterile ultrapure water was used as a second control.

Positive controls:

- purified DNA from bacteria at different dilutions

- pure inactivated bacteria

- insect DNA spiked with bacteria cells.

Positive controls were used in PCR plates distinct from those used for the analysis of insect samples.

**Appendix 3**

Avramov Z., Ivanova I. & Laginova M. (2011) Screening for phytoplasma presence in leafhoppers and planthoppers collected in Bulgarian vineyards. *Bulletin of Insectology*, 64, S115-S116

Busby J.R. (1991) BIOCLIM: a bioclimate analysis and prediction system. *Plant Protection Quarterly*, 6, 8-9

Carpenter G., Gillison A.N. & Winter J. (1993) DOMAIN: a flexible modelling procedure for mapping potential distributions of plants and animals. *Biodiversity and conservation*, 2, 667-680

Chudzicka E. (1989) Leafhoppers (Homoptera, Auchenorrhyncha) of moist meadows on the Mazo Vian lowland. *Memorabilia Zoologica*, 43, 93-118

Cvrković T., Jović J., Mitrović M., Krstić O., Krnjajić S. & Toševski I. (2011) Potential new hemipteran vectors of stolbur phytoplasma in Serbian vineyards. *Bulletin of Insectology*, 64, S129-S130

D’Urso V. & Mifsud D. (2012) A preliminary account of the Auchenorrhyncha of the Maltese Islands (Hemiptera). *Bulletin of the Entomological Society of Malta*, 5, 57-72

Fereres A. (2016) Estudios sobre los vectores transmisores de *Xylella fastidiosa* en olivar. *Jornada de Divulgación Proyecto PONTE 14 de Diciembre. Madrid*

Guglielmino A., Bückle C. & Remane R. (2005) Contribution to the knowledge of the Auchenorrhyncha fauna of Central Italy (Hemiptera, Fulgoromorpha et Cicadomorpha). *Marburger Entomologische Publikationen*, 3, 13-98

Günthart H. (1990) Hom. Auchenorrhyncha, collected in Istria Yugoslavia in spring 1974. *Scopolia Supplementum*, 1, 97-99

Halkka A., Vilbaste J. & Raatikainen M. (1980) Colour gene allele frequencies correlated with altitude of habitat in *Philaenus* populations. *Hereditas*, 92, 243-246

Holzinger v.W.E. (1996) Die Zikadenfauna wärmeliebender Eichenwälder Ostösterreichs (Insecta: Homoptera, Auchenorrhyncha). *Mitteilungen des Naturwissenschaftlichen Vereines für Steiermark*, 126, 169-187

Ivanauskas A., Valiunas D., Ivinskis P. & Rimsaite J. (2014) Some data on Cicadomorpha and Fulgoromorpha (Insecta, Hemiptera) of Lithuania. *New and Rare for Lithuania Insect Species*, 26, 26-30

Karadjova O. & Krusteva H. (2016) Species composition and population dynamics of the harmful insect fauna (Hemiptera: Cicadomorpha, Fulgoromorpha and Sternorrhyncha) of winter triticale. *Bulgarian Journal of Agricultural Science*, 22, 619-626

Krnjajic S.B. (2008) The role of the cicada Scaphoideus titanus Ball in transmitting the grapevine golden yellowing phytoplasma (Flavescence doree). [Doctoral dissertation].

Kula E. (2002) The leafhopper fauna in birch (*Betula pendula* Roth) stands. *Journal of Forest Science*, 48, 351–360

Lecheva I., Karova A. & Markin G.P. (2008) The insect fauna of Chondrilla juncea L.(Asteraceae) in Bulgaria and preliminary studies of Schinia cognate (L.)(Lepidoptera: Noctuidae) as a potential biological control agent. In: *Proceedings of the XII International Symposium on Biological Control of Weeds* pp. 301-305. CABI

Lis A., Maryańska-Nadachowska A., Lachowska-Cierlik D. & Kajtoch Ł. (2014) The secondary contact zone of phylogenetic lineages of the Philaenus spumarius (Hemiptera: Aphrophoridae): an example of incomplete allopatric speciation. *Journal of Insect Science*, 14

Lodos N. & Kalkandelen A. (1981) Preliminary list of Auchenorrhyncha with notes on distribution and importance of species in Turkey. VI. Families Cercopidae and Membracidae. *Turkish Journal of Entomology*, 5, 133-149

Loukas M. & Drosopoulos S. (1992) Population genetics of the spittlebug genus *Philaenus* (Homoptera: Cercopidae) in Greece. *Biological Journal of the Linnean Society*, 46, 403-413

Malenovský I., Bückle C., Guglielmino A., Koczor S., Nickel H., Seljak G., Schuch S. & Witsack W. (2013) Contribution to the Auchenorrhyncha fauna of the Pálava Protected Landscape Area (Czech Republic) (Hemiptera: Fulgoromorpha et Cicadomorpha). *Cicadina*, 13, 29-41

Maryańska-Nadachowska A., Kajtoch L. & Lachowska D. (2012) Genetic diversity of *Philaenus spumarius* and *P. tesselatus* (Hemiptera, Aphrophoridae): Implications for evolution and taxonomy. *Systematic Entomology*, 37, 55-64

Petrova V., Jankevica L. & Samsone I. (2013) Species of Phytophagous Insects Associated with Strawberries in Latvia. *Proceedings of the Latvian Academy of Sciences. Section B. Natural, Exact, and Applied Sciences*, 67, 124-129

Radović M. & Pešić V. (2014) Contribution to the knowledge on Cicadomorpha (Hemiptera: Auchenorrhyncha) of Montenegro. *Ecologica Montenegrina*, 1, 113-116

Riedle-Bauer M., Tiefenbrunner A. & Tiefenbrunner W. (2006) Untersuchungen zur Zikadenfauna (Hemiptera, Auchenorrhyncha) einiger Weingärten Ostösterreichs und ihrer nahen Umgebung. *Linzer Biologische Beiträge*, 38, 1637-1654

Rodrigues A.S.B., Silva S.E., Marabuto E., Silva D.N., Wilson M.R., Thompson V., Yurtsever S., Halkka A., Borges P.A.V., Quartau J.A., Paulo O.S. & Seabra S.G. (2012) New mitochondrial and nuclear evidences support recent demographic expansion and an atypical phylogeographic pattern in the spittlebug *Philaenus spumarius* (Hemiptera, Aphrophoridae). *PlosOne*, 9, e98375

Seabra S.G., Pina-Martins F., Marabuto E., Yurtsever S., Halkka O., _Quartau J.A. & Paulo O.S. (2010) Molecular phylogeny and DNA barcoding in the meadow-spittlebug *Philaenus spumarius* (Hemiptera, Cercopidae) and its related species. *Molecular Phylogenetics and Evolution*, 56, 462-467

Stoikou M.G. & Karanikola P.P. (2014) The entomofauna on the leaves of two forest species, Fagus sylvatica and Corylus avelana, in Menoikio Mountain of Serres. *Entomologica Hellenica*, 23, 65-73

Świerczewski D. & Błaszczyk J. (2010) Fauna piewików (Hemiptera: Fulgoromorpha et Cicadomorpha) Parku Krajobrazowego „Stawki”. *Acta Entomologica Silesiana*, 18, 9-22

**Figure S1.**

**
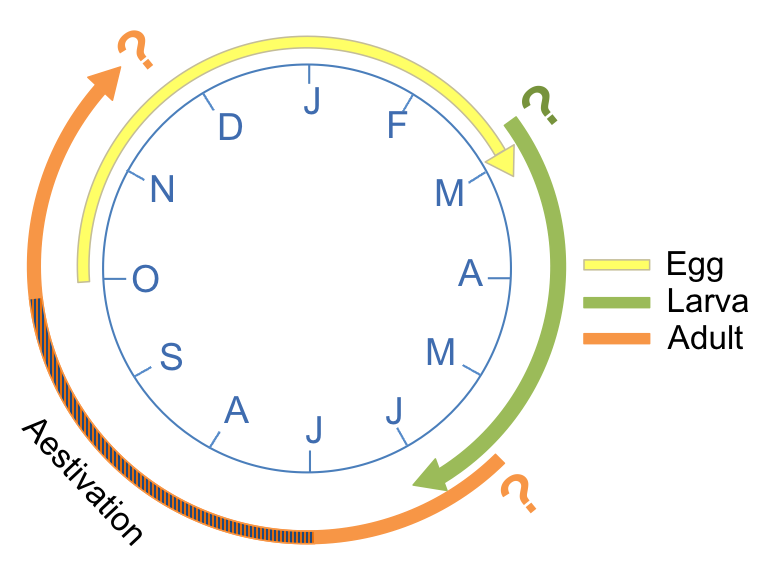
**

**Figure S2**

**
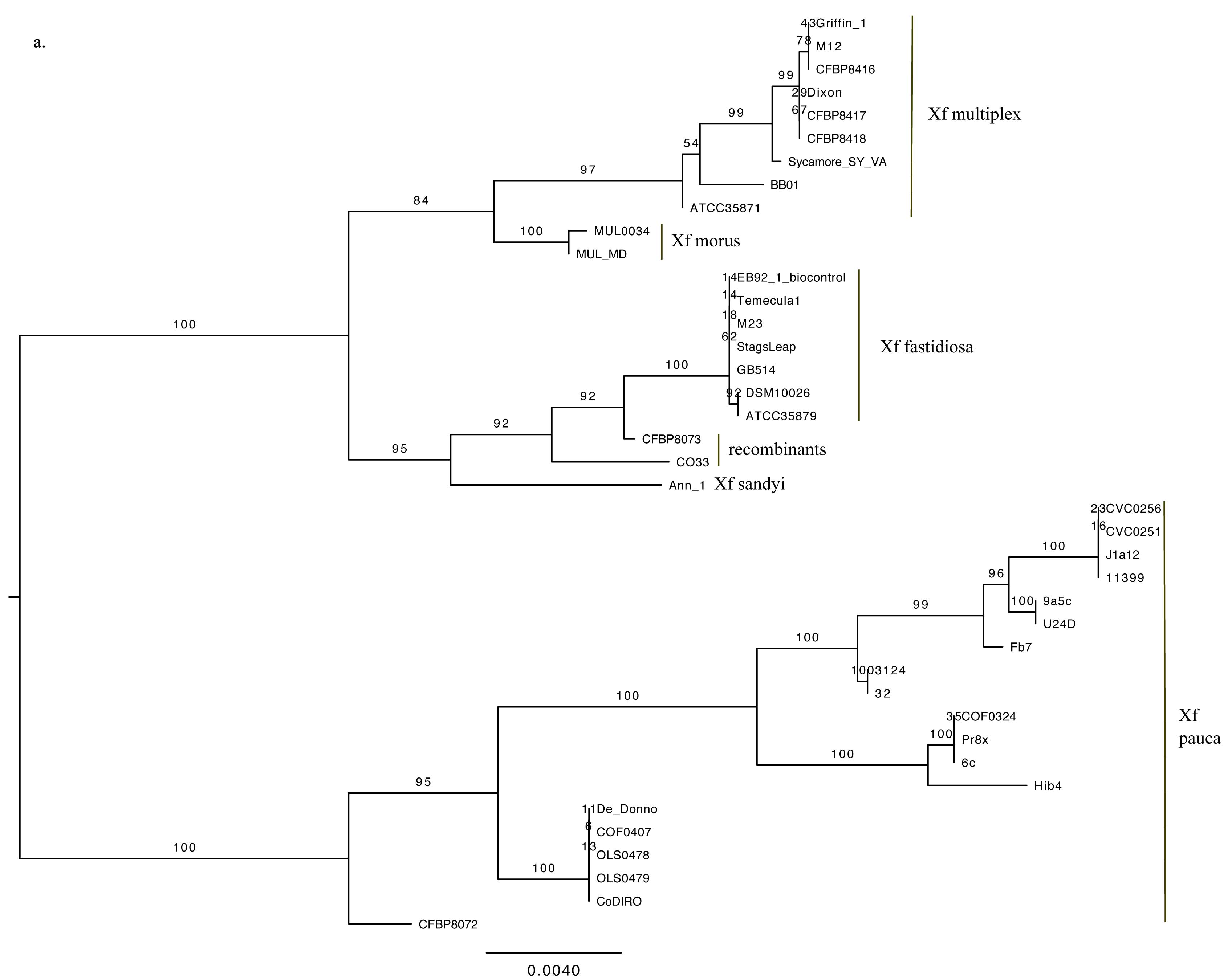

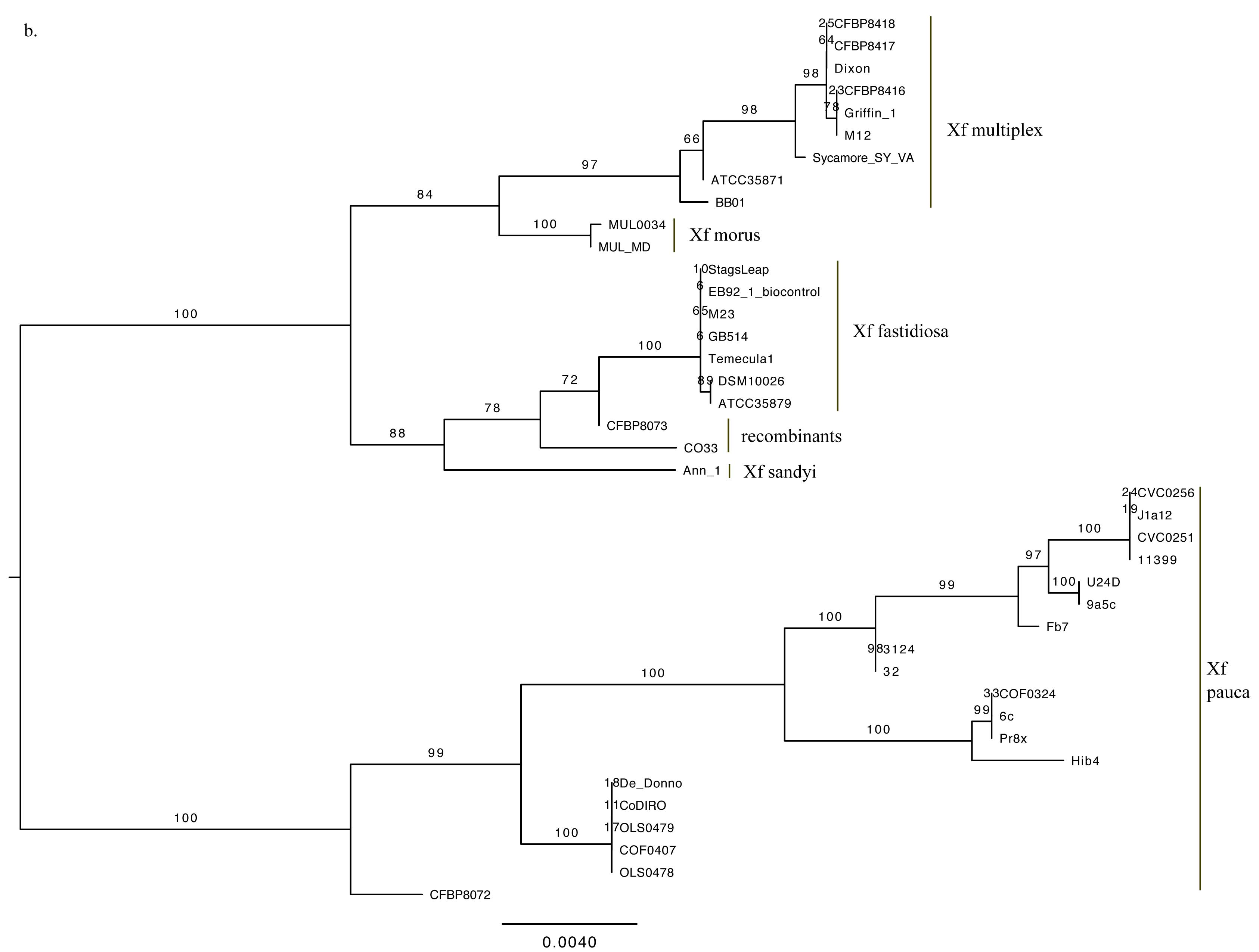
**

**Figure S3**

**
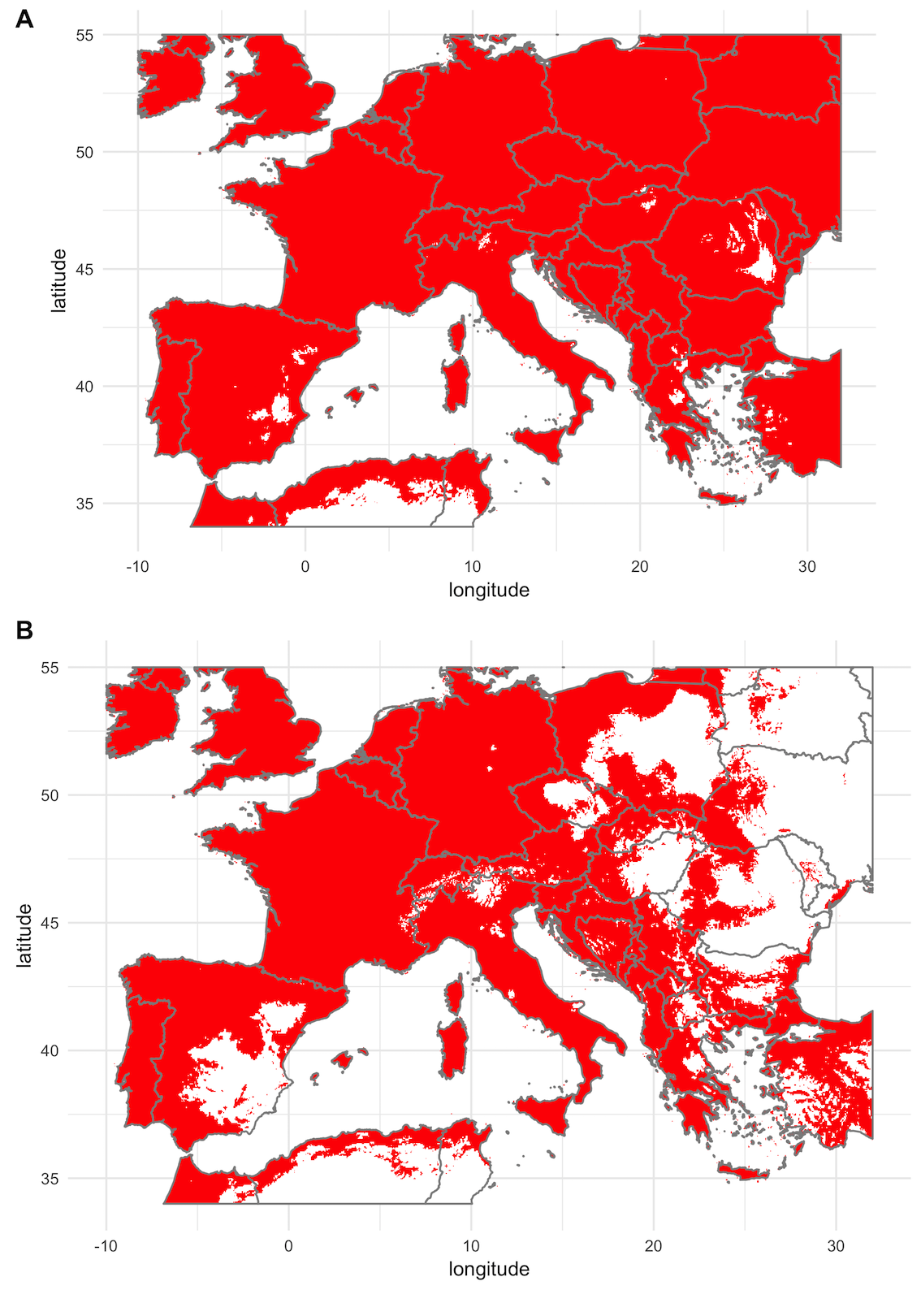
**

**Table S1.**

| **code vial** | **Sampling date** | **locality** | **collected by** | **included in molecular tests** |
| --- | --- | --- | --- | --- |
| JRAS06828 | 06/06/2016 | Albitreccia, Bocca di Belle Valle | Cruaud A., Rasplus J.-Y. | yes (site A on Fig. 3) |
| JSTR03702 | 22/10/2016 | Albitreccia, Bocca di Belle Valle | Godefroid M., Streito J.-C., Thuillier J.-M. | yes (site A on Fig. 3) |
| JRAS06831 | 07/06/2016 | Road to Linguizzetta | Cruaud A., Rasplus J.-Y. | yes (site B on Fig. 3) |
| JRAS06835 | 08/06/2016 | Porto-Vecchio, Route de Piccovaggia | Cruaud A., Rasplus J.-Y. | yes (site C on Fig. 3) |
| JSTR03644 | 20/10/2016 | Porto-Vecchio, Route de Piccovaggia | Godefroid M., Streito J.-C., Thuillier J.-M. | yes (site C on Fig. 3) |
| JRAS06838 | 11/06/2016 | Zigliara, Bains de Taccana, 1.3km S | Cruaud A., Rasplus J.-Y. | yes (site D on Fig. 3) |
| JSTR03686 | 21/10/2016 | Zigliara, Bains de Taccana, 1.3km S | Godefroid M., Streito J.-C., Thuillier J.-M. | yes (site D on Fig. 3) |
| JRAS06849 | 07/06/2016 | nr Abbazia | Cruaud A., Rasplus J.-Y. | yes (site E on Fig. 3) |
| JSTR03669 | 21/10/2016 | Belvédère-Campomoro, Campomoro, l'Uomo | Godefroid M., Streito J.-C., Thuillier J.-M. | yes (site F on Fig. 3) |
| JSTR03697 | 21/10/2016 | Olmeto, Abbartello, Plage de Tenutella | Streito J.-C. | yes (site G on Fig. 3) |
| JSTR03725 | 23/10/2016 | Piana, Monte San Ghiabicu | Streito J.-C. | yes (site H on Fig. 3) |
| JSTR03740 | 27/10/2016 | Calvi, Hotel Mariana | Streito J.-C. | yes (site I on Fig. 3) |
| MGOD00145 | 17/06/2016 | Corte, Gorges de la Restonica | Godefroid M., Streito J.-C. | yes (site J on Fig. 3) |
| MGOD00159 | 21/06/2016 | Salice, Bocca di Tartavellu | Godefroid M., Streito J.-C. | yes (site K on Fig. 3) |
| JRAS06846 | 06/06/2016 | nr Porticcio | Cruaud A., Rasplus J.-Y. | no |
| JRAS06848 | 08/06/2016 | nr Aleria | Cruaud A., Rasplus J.-Y. | no |
| JRAS06850 | 08/06/2016 | Solaru | Cruaud A., Rasplus J.-Y. | no |
| JRAS06851 | 08/06/2016 | Fautea | Cruaud A., Rasplus J.-Y. | no |
| JRAS06853 | 10/06/2016 | nr Caldarello | Cruaud A., Rasplus J.-Y. | no |
| JRAS06856 | 10/06/2016 | Pietra Rossa | Cruaud A., Rasplus J.-Y. | no |
| JRAS06857 | 10/06/2016 | N Serra-di-Ferro | Cruaud A., Rasplus J.-Y. | no |
| JRAS06858 | 10/06/2016 | N Serra-di-Ferro | Cruaud A., Rasplus J.-Y. | no |
| JSTR02972 | 16/06/2016 | Aléria, Ruale | Streito J.-C. | no |
| JSTR03022 | 17/06/2016 | Corte, Gorges de la Restonica | Streito J.-C. | no |
| JSTR03063 | 18/06/2016 | Calvi, Hotel Mariana | Streito J.-C. | no |
| JSTR03119 | 21/06/2016 | Vero | Streito J.-C. | no |
| JSTR03130 | 21/06/2016 | Azzana; Pont d'Azzana | Streito J.-C. | no |
| JSTR03143 | 21/06/2016 | Vero | Streito J.-C. | no |
| JSTR03163 | 22/06/2016 | Grosseto-Prugna, Tour Capitello, Estuaire de la Gravona | Streito J.-C. | no |
| JSTR03518 | 17/10/2016 | Ersa, Tollare | Godefroid M., Streito J.-C., Thuillier J.-M. | no |
| JSTR03519 | 17/10/2016 | Morsiglia, Mucchietia | Godefroid M., Streito J.-C., Thuillier J.-M. | no |
| JSTR03531 | 17/10/2016 | Luri, Santa Severa | Godefroid M., Streito J.-C., Thuillier J.-M. | no |
| JSTR03541 | 17/10/2016 | Brando, Tour de Sacro | Godefroid M., Streito J.-C., Thuillier J.-M. | no |
| JSTR03550 | 17/10/2016 | Ville-di-Pietrabugno, Monte Pinzutu | Godefroid M., Streito J.-C., Thuillier J.-M. | no |
| JSTR03558 | 17/10/2016 | Bastia | Godefroid M., Streito J.-C., Thuillier J.-M. | no |
| JSTR03566 | 18/10/2016 | Furiani, Allée Santa Barbara | Godefroid M., Streito J.-C., Thuillier J.-M. | no |
| JSTR03572 | 18/10/2016 | Furiani, Allée des Arbousiers | Godefroid M., Streito J.-C., Thuillier J.-M. | no |
| JSTR03585 | 18/10/2016 | Aléria, Pietroni | Godefroid M., Streito J.-C., Thuillier J.-M. | no |
| JSTR03602 | 18/10/2016 | Ghisonaccia, Nielluccio | Godefroid M., Streito J.-C., Thuillier J.-M. | no |
| JSTR03607 | 19/10/2016 | Porto-Vecchio, Golfo di Sogno | Godefroid M., Streito J.-C., Thuillier J.-M. | no |
| JSTR03613 | 19/10/2016 | Lecci, San Ciprianu | Godefroid M., Streito J.-C., Thuillier J.-M. | no |
| JSTR03619 | 19/10/2016 | Zonza, Cirendinu | Godefroid M., Streito J.-C., Thuillier J.-M. | no |
| JSTR03623 | 19/10/2016 | Zonza, Cirendinu, Punta Capicciola | Godefroid M., Streito J.-C., Thuillier J.-M. | no |
| JSTR03629 | 19/10/2016 | Zonza, Vallicone | Godefroid M., Streito J.-C., Thuillier J.-M. | no |
| JSTR03634 | 19/10/2016 | Zonza, Cappiciola | Godefroid M., Streito J.-C., Thuillier J.-M. | no |
| JSTR03636 | 19/10/2016 | Zonza, Padulu Tortu | Godefroid M., Streito J.-C., Thuillier J.-M. | no |
| JSTR03641 | 19/10/2016 | Lecci, Chemin de Mulinacciu | Godefroid M., Streito J.-C., Thuillier J.-M. | no |
| JSTR03657 | 20/10/2016 | Bonifacio, Foce di Lera, T40 | Godefroid M., Streito J.-C., Thuillier J.-M. | no |
| JSTR03658 | 20/10/2016 | Monacia-d'Aullène, Roccapina | Godefroid M., Streito J.-C., Thuillier J.-M. | no |
| JSTR03660 | 20/10/2016 | Sartène, Pero Longo, D250, 2kms NE | Godefroid M., Streito J.-C., Thuillier J.-M. | no |
| JSTR03675 | 21/10/2016 | Belvédère-Campomoro, Portigliolo | Godefroid M., Streito J.-C., Thuillier J.-M. | no |
| JSTR03678 | 21/10/2016 | Propriano, Bartaccia | Streito J.-C. | no |
| JSTR03694 | 21/10/2016 | Pila-Canale, Calzola, 1.5km NE | Godefroid M., Streito J.-C., Thuillier J.-M. | no |
| JSTR03716 | 22/10/2016 | Grosseto-Prugna, Porticcio | Godefroid M., Streito J.-C., Thuillier J.-M. | no |
| JSTR03730 | 24/10/2016 | Ota, Marine de Porto | Streito J.-C. | no |
| JSTR03735 | 26/10/2016 | Palasca, Désert des Agriates, Cima a Forca | Streito J.-C. | no |
| JSTR03739 | 26/10/2016 | Palasca, Désert des Agriates, Punta Liatoggiu | Streito J.-C. | no |
| MGOD00148 | 18/06/2016 | Montegrosso, Montemaggiore | Godefroid M., Streito J.-C. | no |
| MGOD00149 | 18/06/2016 | Calvi | Godefroid M., Streito J.-C. | no |
| MGOD00152 | 19/06/2016 | Lumio | Godefroid M., Streito J.-C. | no |
| MGOD00154 | 19/06/2016 | Muro | Godefroid M., Streito J.-C. | no |
| MGOD00156 | 19/06/2016 | Muro | Godefroid M., Streito J.-C. | no |

**Table S2**

|  |  |  |  |  |
| --- | --- | --- | --- | --- |
| **Target locus** | Primer name | Sequence (5'-3') | Start position (on the target locus without primers) | Product length (primers included) (bp) |
|  |  |  |  |  |
| **leuA** | leuA-NesF37 | CGCGCTGGAAGAACTCACTA | 37 | 649 |
|  | leuA-NesR685 | GGCCCCGATACCAACACTG | 685 |  |
| **petC** | petC-NesF11 | CGTATCGCTGCTGATTTGGG | 11 | 475 |
|  | petC-NesR485 | CCCATACTCTCAAAGAATGCCG | 485 |  |
| **malF** | malF-NesF4 | ATCGGGATGTTCTTCGGTGT | 4 | 726 |
|  | malF-NesR729 | CCACCACTTAAAACCCTCCTCA | 729 |  |
| **cysG** | cysG-NesF29 | GTCCGGCAGCGTCACC | 29 | 554 |
|  | cysG-NesR582 | CAGCCTCACACAGGCGT | 582 |  |
| **nuoL** | nuoL-NesF24 | CGAGTTTCTACAGCTTCCGC | 24 | 515 |
|  | nuoL-NesR538 | CAGAATACGCACCGGCAAC | 538 |  |
| **gltT** | gltT-Nes17F | CCGCTGGGRACCTTTGGATT | 17 | 623 |
|  | gltT-Nes639R | CCCGTTTCTTTTGCCACCAG | 639 |  |
| **holC** | HolC-NesF1 | ATTTGATTKCCAAACCGCGCT | 1 | 378 |
|  | HolC-NesR378 | CGCGTGYCTTGTAGTGTTTC | 378 |  |

To facilitate Sanger sequencing reactions of PCR products, nested-PCR primers with a 5 ' tail can be used.

For primers forward, TailF: TGTAAAACGACGGCCAGT, for primers reverse, TailR: CAGGAAACAGCTATGAC.

**Table S3**

| **PCR1** | Classic MLST | (Yuan et al., 2010) | |  |
| --- | --- | --- | --- | --- |
|  |  |  |  |  |
|  | 50 μL reaction | Final conc. |  |  |
| KAPA Taq Buffer B (10X) 1.5 mM MgCl2 at 1X | 5 μL | 1X |  |  |
| 25 mM MgCl2 | 0.2 μL | 1.6 mM |  |  |
| 10 mM dNTP Mix | 4 μL | 0.2 mM each |  |  |
| 10 μM Forward Primer | 2 μL | 0.4 μM |  |  |
| 10 μM Reverse Primer | 2 μL | 0.4 μM |  |  |
| 5 U/μL KAPA Taq DNA Pol | 0.2 μL | 1 U |  |  |
| Triton X100 1% | 5 μL | 0.10% |  |  |
| PCR-grade water | Up to 40 μL |  |  |  |
| Template DNA (20% of the purified insect DNA) | 10 µl | 1 to 2.5 µg |  |  |
|  |  |  |  |  |
|  |  |  |  |  |
| **PCR2** | Nested |  |  |  |
|  |  |  |  |  |
|  | 50 μL reaction | Final conc. |  |  |
| KAPA Taq Buffer B (10X) 1.5 mM MgCl2 at 1X | 5 μL | 1X |  |  |
| 25 mM MgCl2 | 0.2 μL | 1.6 mM |  |  |
| 10 mM dNTP Mix | 4 μL | 0.2 mM each |  |  |
| 10 μM Forward Primer | 1 μL | 0.2 μM |  | possibly with 5' tailed primers |
| 10 μM Reverse Primer | 1 μL | 0.2 μM |  |  |
| 5 U/μL KAPA Taq DNA Pol | 0.2 μL | 1 U |  |  |
| Triton X100 1% | 5 μL | 0.10% |  |  |
| PCR-grade water | Up to 45 μL |  |  |  |
| Template DNA (10% of the non-purified PCR1) | 5 µl |  |  |  |
|  |  |  |  |  |
| KAPA Taq PCR Kit, ref KK1015 | |  |  |  |
|  |  |  |  |  |
| KAPA Taq Buffer B is a Tris-potassium chloride buffer | | |  |  |
| Buffer B is recommended for applications where high sensitivity is required | | | | |
| (e.g. when the template is limiting) | |  |  |  |

**Reference cited**

Yuan, X., Morano, L., Bromley, R., Spring-Pearson, S., Stouthamer, R., Nunney, L., 2010. Multilocus sequence typing of *Xylella fastidiosa* causing Pierce's disease and oleander leaf scorch in the United States. Phytopathology 100, 601-611.

**Table S4**

|  | **PCR1 Triplex** | MLST Triplex |  |  |  |
| --- | --- | --- | --- | --- | --- |
|  |  |  |  |  |  |
|  |  | 50 μL reaction | Final conc. |  |  |
|  | KAPA Taq Buffer B (10X) 1.5 mM MgCl2 at 1X | 5 μL | 1X |  |  |
|  | 25 mM MgCl2 | 0.2 μL | 1.6 mM |  |  |
|  | 10 mM dNTP Mix | 4 μL | 0.2 mM each |  | Triplex 1 |
| target 1 | 10 μM Forward Primer | 1 μL | 0.2 μM |  | *gltT* + leuA + *petC* |
|  | 10 μM Reverse Primer | 1 μL | 0.2 μM |  |  |
| target 2 | 10 μM Forward Primer | 1 μL | 0.2 μM |  | Triplex 2 |
|  | 10 μM Reverse Primer | 1 μL | 0.2 μM |  | *cysG* + *malF* + *nuoL* |
| target 3 | 10 μM Forward Primer | 1 μL | 0.2 μM |  |  |
|  | 10 μM Reverse Primer | 1 μL | 0.2 μM |  |  |
|  | 5 U/μL KAPA Taq DNA Pol | 0.2 μL | 1 U |  |  |
|  | Triton X100 1% | 5 μL | 0.10% |  |  |
|  | PCR-grade water | Up to 40 μL |  |  |  |
|  | Template DNA (20% of the purified insect DNA) | 10 µl | 1 to 2.5 µg |  |  |
|  |  |  |  |  |  |
|  |  |  |  |  |  |
|  | **PCR2** | Nested |  |  |  |
|  |  |  |  |  |  |
|  |  | 50 μL reaction | Final conc. |  |  |
|  | KAPA Taq Buffer B (10X) 1.5 mM MgCl2 at 1X | 5 μL | 1X |  |  |
|  | 25 mM MgCl2 | 0.2 μL | 1.6 mM |  |  |
|  | 10 mM dNTP Mix | 4 μL | 0.2 mM each |  |  |
| One of the target | 10 μM Forward Primer | 1 μL | 0.2 μM |  | possibly with 5' tailed primers |
| from PRC1 Triplex | 10 μM Reverse Primer | 1 μL | 0.2 μM |  |  |
|  | 5 U/μL KAPA Taq DNA Pol | 0.2 μL | 1 U |  |  |
|  | Triton X100 1% | 5 μL | 0.10% |  |  |
|  | PCR-grade water | Up to 45 μL |  |  |  |
|  | PCR1 (10% of the non-purified PCR1 Triplex) | 5 µl |  |  |  |

**Table S5**

|  |  | |  |
| --- | --- | --- | --- |
|  |  |  |  |
| Step | Temperature | Duration | Cycles |
| Initial denaturation | 94°C | 4 min | 1 |
| Denaturation | 94°C | 30 sec | 12, - 0,7°C/cycle |
| Annealing | 65°C* | 1 min |  |
| Extension | 72°C | 1 min |  |
| Denaturation | 94°C | 30 sec | 30 |
| Annealing | 56°C | 1 min |  |
| Extension | 72°C | 1 min |  |
| Final extension | 72°C | 5 min | 1 |
| Hold | 14°C | ∞ | 1 |

*TouchDown PCR from 65°C to 56°C
